# Antistress Effects of Terpinen-4-ol and Compounds of Mimicked Yuzu Synthetic Fragrance in Humans and Mice

**DOI:** 10.3390/foods13193051

**Published:** 2024-09-25

**Authors:** Takuma Kitamoto, Takafumi Mizushige, Xiaonan Xie, Taisei Uematsu, Risako Ogura, Kakeru Sato, Yuki Yamazaki, Tsubasa Matsushita, Hiroshi Hasegawa

**Affiliations:** 1 School of Engineering, Utsunomiya University, Utsunomiya 321-8585, Tochigi, Japan; kitamoto@is.utsunomiya-u.ac.jp (T.K.); sato.k@sound.is.utsunomiya-u.ac.jp (K.S.); yamazaki.y@sound.is.utsunomiya-u.ac.jp (Y.Y.); matsushita.t@sound.is.utsunomiya-u.ac.jp (T.M.); 2 School of Agriculture, Utsunomiya University, Utsunomiya 321-8505, Tochigi, Japan; mizushige@cc.utsunomiya-u.ac.jp (T.M.); a173206@cc.utsunomiya-u.ac.jp (T.U.); mc226693@s.utsunomiya-u.ac.jp (R.O.); 3 Center for Bioscience Research and Education, Utsunomiya University, Utsunomiya 321-8505, Tochigi, Japan; xie@cc.utsunomiya-u.ac.jp; 4 School of Data Science and Management, Utsunomiya University, Utsunomiya 321-8505, Tochigi, Japan

**Keywords:** yuzu, synthetic fragrance, antistress, terpinen-4-ol

## Abstract

This study investigated the antistress effects of yuzu synthetic fragrances by employing three experiments on humans and mice using two yuzu synthetic fragrances and five single compounds. We prepared two synthetic fragrances based on the component analysis of two natural yuzu essential oils extracted by cold-pressed and steam-distilled extraction methods. Chromogranin A (CgA) and heart rate (HR) were used as stress indices in human experiments. Immobility time during the forced swim test was used as a stress index in mice experiments. We analyzed brain mechanisms by measuring the expression of neurotrophic factors, brain-derived neurotrophic factor (BDNF), nerve growth factor (NGF), and neurotrophin-3 (NT-3) in the mice experiments. Synthetic yuzu fragrance mimicked steam-distilled oil (SD) significantly reduced participants’ heart rate in experiment 1. In the forced swim test conducted in experiment 2, SD significantly reduced immobility time, and increased the expression of neurotrophic factors BDNF, NGF, and NT-3 in the hippocampus of mice. In experiment 3, focusing on single compounds, terpinen-4-ol significantly reduced immobility time in the forced swim test. These findings indicate that inhalation of SD and terpinen-4-ol has antistress effects. Terpinen-4-ol is a strong candidate for further investigation as a potential stress-reducing agent.

## 1. Introduction

Chronic stress is a significant risk factor for the development of various health conditions, including hypertension, atherosclerosis, nonalcoholic fatty liver disease, and psychiatric disorders such as depression [1,2]. Depression is a common mental disorder and about 280 million people suffer from depression worldwide. Depression is a suicidal illness, and more than 700 thousand people die by suicide each year [3]. The stress response, the main trigger for the onset of depression, is regulated in the brain, with the hippocampus and prefrontal cortex being particularly important brain regions for the stress response [4].

A fragrance has a balancing effect on the body and mind. In particular, natural essential oils are used in aromatherapy to enhance physical and mental well-being. This is because the complex aroma of natural essential oils, which contain dozens to hundreds of aromatic components, is thought to influence physiological and psychological states [5,6]. On the other hand, artificially prepared fragrances (synthetic fragrances) are generally used for daily products such as detergents, air fresheners, fabric softeners, and flavoring for foods and beverages. Synthetic fragrances are more stable and inexpensive than natural fragrances, although they are more monotonous and less variable in flavor.

Yuzu (*Citrus junos* Sieb. ex Tanaka) is an aromatic citrus familiar to Japanese people. People enjoy yuzu citrus for its refreshing aroma, in contrast to oranges and tangerines, which are enjoyed for their taste. In recent years, yuzu has been actively exported to Europe, the United States, and other parts of the world, and is expected to continue expanding worldwide [7,8]. Some experiments have examined the antistress effects of natural yuzu essential oil. For example, Matsumoto reported that the aroma of natural yuzu essential oil might relieve negative emotional stress and inhibit sympathetic nervous system activity [9]. Ohata et al. reported that inhaling yuzu essential oil aroma may induce parasympathetic dominance, indicated by physiological changes, and reduced subjective fatigue, alongside increased feelings of refreshment [10].

Multiple techniques exist for essential oil extraction, including cold pressing, water distillation, steam distillation, and solvent extraction. For citrus fruits, cold-pressing and steam-distillation methods have been the dominant methods due to their simplicity and relatively inexpensive equipment requirements [11]. The chosen extraction method can influence the composition of compounds within the essential oil [12]. Therefore, incorporating the extraction method as a design consideration is crucial for research on the functionality of plant-derived fragrances.

This study aims to develop a more stable and inexpensive synthetic fragrance similar to the characteristic aroma of natural essential oils and to clarify the antistress effects of these fragrances from the perspective of human physiology, mouse ethology, and mouse cranial nerves. For this purpose, we prepared two types of yuzu synthetic fragrances that reproduce the aroma of natural essential oils. We then investigated their antistress effects by human chromogranin A (CgA) and heart rate (HR) analysis, mouse forced swim test, and nerve growth factor analysis. We finally report on the antistress effects of the yuzu synthetic fragrance obtained in this study.

## 2. Materials and Methods

### 2.1. Stimuli

For this study, we developed two yuzu synthetic fragrances. These synthetic fragrances were prepared based on gas chromatography–mass spectrometry (GC/MS) analysis data of natural essential oils extracted using two different methods. This section details the extraction of the natural fragrances, the GC/MS analysis, and the preparation of the yuzu synthetic fragrances.

#### 2.1.1. Extract Natural Essential Oils from Yuzus

We prepared two synthetic fragrances based on the component analysis of two natural yuzu essential oils extracted by different extraction methods. Yuzu, a raw material for natural essential oil, was harvested in Tochigi Prefecture, Japan. We extracted essential oils from these yuzus by cold-pressing and steam-distillation methods. The cold pressing method extracts the essential oil components contained in the vacuoles of the flavedo by squeezing the yuzu peel by hand [13]. The steam-distillation method extracts essential oil by vaporizing volatile compounds contained in the yuzu peel by generating steam from the bottom of the yuzu peel. The yuzu essential oil via steam distillation was extracted by a specialized extraction company.

#### 2.1.2. GC/MS Analysis

We employed GC/MS to identify compounds in essential oils. The GC/MS is an Agilent 7890A gas chromatograph equipped with an Agilent PAL3 autosampler and coupled to an Agilent 5977B mass selective detector (Agilent Technologies, Santa Clara, CA, USA). The column was a DB-HeavyWAX (30 + 10 m guard column, 0.25 mm i.d., film thickness of 0.25 μm; Agilent Technologies, Santa Clara, CA, USA). We employed headspace analysis to measure the volatile compounds of yuzu in a state as close as possible to the scent we perceived. We injected each sample in 0.5 mL in a split mode (1:10). The injector temperature was 250 °C, and the carrier helium gas flow rate was 1.2 mL/min. The oven temperature was kept at 50 °C for 1 min and increased to 260 °C at a rate of 8 °C/min. The final temperature was held at 260 °C for 1 min. The mass spectrometer was used at the ionization voltage of 70 eV (EI) and ion source temperature of 230 °C. Retention indices of the compounds were calculated from the retention times of n-alkanes mix C4–30 (Hayashi Pure Chemical Ind., Ltd., Osaka, Japan). The retention indices (RI) data are shown in Table S1 and the corresponding total ion chromatogram (TIC) data are in Figure S1.

#### 2.1.3. Synthetic Yuzu Fragrances and Single Compounds

The chemical compounds in cold-pressed and steam-distilled essential oils identified by GC/MS analysis are listed in Tables S2 and S3. Based on the compounds, a perfumer prepared two synthetic fragrances that mimicked cold-pressed oil (CP) and steam-distilled oil (SD). These fragrances were prepared by the perfumer from compounds available as fragrances at the perfumer’s judgment. The prepared compounds and the content rate are shown in Table 1. Limonene, γ-Terpinene, Linalool, ß-Pinene, Terpinen-4-ol, α-Terpineol, *trans*-2-Hexenal, Camphene and Octanal were obtained from Toyotama International Inc. (Tokyo, Japan). p-Cymene, ß-Myrcene, α-Pinene, ß-Ocimene and Hexanal were obtained from Aroma Pyxis Co., Ltd. (Saitama, Japan). Terpinolene, Thymol and Ethyl Acetate were obtained from Daiho Perfumery Co., Ltd. (Osaka, Japan). *cis*-3-Hexenyl formate and *trans*-ß-Farnesene were were obtained from Fragrance House Ltd. (Tokyo, Japan). Dipropylene Glycol was obtained from Adeka chemical supply corporation (Tokyo, Japan). We used the synthetic fragrances CP and SD as stimuli in experiments 1 and 2. We also used the five single compounds included in SD but not in CP as stimuli in experiment 3.

### 2.2. Experiment 1: Antistress Effect of Fragrance under Mental Stress Load with Human Participants

This experiment aimed to investigate the effects of synthetic yuzu fragrances on physiological responses in humans, as measured by CgA and HR.

#### 2.2.1. Participants

We recruited 34 participants (16 males and 18 females; M_age_ = 22.1, SD_age_ = 1.4), and divided them into three groups: a cold-pressed group, a steam-distillation group, and a control group. The participants in the cold-pressed and steam-distillation groups had passed an olfactory test using a standard odor kit (Daiichi Yakuhin Sangyo Co., Ltd., Tokyo, Japan).

To minimize the influence of physical state and other external factors, participants were asked in advance to have at least 7 h of sleep and avoid overeating or overdrinking on the day before the experiment. Likewise, participants were asked to abstain from using scented hairstyling products or wearing perfume on the day of the experiment, from eating within two hours of the experiment, and from hydration or smoking within a half hour of the experiment.

#### 2.2.2. CgA Concentration

CgA is an acidic glycoprotein isolated from within the chromaffin granules of the adrenal medulla and is widely distributed in the endocrine and nervous systems [14,15]. CgA is also present in submandibular gland conduits and is released into saliva by autonomic nervous activity when mental stress is applied, resulting in a high CgA concentration, which has been used as an indicator of mental stress [9,16]. In the present study, we calculated CgA concentrations from saliva collected before and after a simple calculation task, which is a stressor, to examine the effects of mental stress.

#### 2.2.3. Heart Rate

Enhancement of sympathetic nerve activity causes an increase in HR, and enhancement of parasympathetic activity causes a decrease in HR [17]. We thus used HR (bpm) as an indicator of stimulative or sedative effects in this study. We attached a portable HR monitor (M200, Polar Electro, Kempele, Finland) to the participant’s non-dominant wrist. HR was measured at 1-second intervals using the monitor while the calculation task was performed.

#### 2.2.4. Procedure

The experiments took place in booths approximately 85 cm wide, 85 cm long, and 75 cm high, enclosed on all sides by transparent sheets, to control the density and diffusion of aroma. For the tasks involving the cold-pressed and steam-distillation groups, we filled the booth with several yuzu aromas using an aroma diffuser (Aromore, Tree of Life Co., Ltd., Tokyo, Japan). We set the temperature of the laboratory at 22 ± 1 °C and the humidity at 50 ± 5%. To minimize the influence of circadian rhythm, we conducted the experiment between 10 a.m. and 1 p.m. [18].

This study has been approved by the Research Ethics Committee of Utsunomiya University (Approval No. H20-0053) and was conducted according to the principles of the Declaration of Helsinki. We first provided details of the experiment to the participants and obtained their written informed consent to participate. After checking their physical condition verbally, we conducted a smell test on the participants. We then conducted an olfactory test on the participants. Participants were asked to rinse their mouths with ultrapure water to clean their intraoral. Afterward, each participant entered the booth and rested by sitting in a chair for 10 min. After resting, samples of the participants’ saliva were collected using the Salisoft^®^ saliva collection devices (Sarstedt Co., Ltd., Rommelsdorf, Germany). The Salisoft^®^ is a device that contains a cotton-like saliva absorber. Participants placed the swab in their mouth and chewed it for 2 min. We then began to present yuzu fragrances to the fragrance environment groups, and vaporized pure water to the control group. Each participant then performed a continuous task involving addition calculation for 30 min as a mental stressor [19]. They were asked to perform as many calculations as possible within the time limit. Immediately after completing this task, participants again collected their saliva using the same method. We conducted this procedure for all 34 participants. The sampling tubes were stored at under −20 °C. Salivary CgA concentrations were determined using the Human Chromogranin A EIA Kit (YK070, Yanaihara Institute, Inc., Shizuoka, Japan) according to the manufacturer’s instructions.

### 2.3. Experiment 2: Forced Swim Test Using Synthetic Fragrances and Gene Expression Measurement in Mice

In the study investigating the antistress effects of synthetic yuzu fragrances, we subjected mice to two forced swim tests. Following the second test, we analyzed gene expression in their brains.

#### 2.3.1. Animals

Mice used in this experiment were raised according to a method previously described [20]. Briefly, male 5-week-old ddY mice (Japan SLC, Inc., Shizuoka, Japan) were raised in plastic cages in an environment-controlled room under a 12-h light–dark cycle at a constant temperature (23 ± 1 °C) and humidity (50 ± 10%). Animals were group-housed for five days to acclimate them to the environment and were provided regular tap water and commercial solid chow (MF; Oriental Yeast, Osaka, Japan) ad libitum. This study was conducted in accordance with the ethical guidelines of the Utsunomiya University Animal Experimentation Committee and in complete compliance with the National Institutes of Health: Guide for the Care and Use of Laboratory Animals. Protocols for the animal experiments were approved by the Utsunomiya University Animal Experimentation Committee (Approval No. A20-0003).

#### 2.3.2. Forced Swim Test

The forced swim test was devised by Porsolt to assess potential antidepressant effects by observing stress-induced depressive behavior in mice. Porsolt proposed that reduced immobility time in the forced swim test signifies an antidepressant-like effect. We conducted an experiment to evaluate the antistress effects of the forced swimming test using immobility time as an indicator [21]. The forced swim test was performed as previously described [20]. Briefly, mice were individually forced to swim in an open cylindrical container (diameter, 10 cm; height, 20 cm) containing 10 cm of water at 25 ± 1 °C. The water was changed after each trial. The forced swim test was performed during the light phase of the light/dark cycle. The total immobility time (s) was measured during a single 6 min test session. Mice were immobile when they made no attempt to escape as reflected by their movements to keep their heads above the water. A decrease in the immobility time was an indicator of an antistress effect.

#### 2.3.3. Procedure

We conducted the forced swim test twice to investigate the antistress effect of the synthetic fragrances. During the first forced swim test, immobility time was measured; neurotrophic factors were assessed in the second test.

After five days of acclimatization, mice were divided into the required number of groups. In the first forced swim test, mice were divided into three groups: a group presented with CP, a group presented with SD, and a control group presented with triethyl citrate. Each synthetic fragrance, which was mixed in triethyl citrate at a concentration of 0.1%, was placed in two corners inside the box (125 μL/m^3^). Under the same conditions, we used triethyl citrate as a control. Each mouse spent 30 min in the box for 3 consecutive days. The forced swim test was started immediately after the 3-day sniffing period.

In the second forced swim test, we investigated the antistress effect of SD based on neurotrophic factors. After five days of acclimatization, mice were divided into two groups: a group presented with SD and a control group presented with triethyl citrate. The mice were euthanized by decapitation, and the hippocampus and prefrontal cortex were collected. The hippocampus and prefrontal cortex were stored at under −20 °C until the measurement of gene expression was performed.

#### 2.3.4. Measurement of Gene Expression

The measurement procedures, reagents, and equipment in this section are the same as in the previously described study [20]. Each hippocampus and prefrontal cortex was homogenized for extracting total RNA using QIAzol Lysis Reagent (QIAGEN Sciences Inc., Germantown, MD, USA) following the manufacturer’s instructions, and purified using the RNeasy Mini Kit (QIAGEN Sciences Inc., Germantown, MD, USA). cDNA was synthesized from mRNA using the ReverTra Ace^®^ qPCR RT Master Mix with the gDNA Remover (Toyobo Co., Osaka, Japan). In quantitative PCR, cDNA was amplified using the LightCycler^®^ 96 System (Roche Diagnostics Co., Mannheim, Germany) with the THUNDERBIRD^®^ qPCR Mix (Toyobo Co., Osaka, Japan) and specific primers for mouse brain-derived neurotrophic factor (BDNF), nerve growth factor (NGF), neurotrophin-3 (NT-3), and β-actin according to the manufacturer’s instructions. These primer sequences are shown in Table 2. Reactions were cycled 45 times with denaturation at 95 °C for 10 s, followed by annealing and elongation at 65 °C for 60 s. The relative expression level of each mRNA was normalized against the mRNA expression level of β-actin.

### 2.4. Experiment 3: Forced Swim Test in Mice Exposed to a Single Compound

What experiments 1 and 2 had in common was that SD had an antistress effect. To identify potential antistress agents, we conducted the forced swim test to evaluate the effects of five single compounds found only in the SD formulation, as compared to the CP formulation.

#### 2.4.1. Animals

In this experiment, mice were raised under the same conditions as those used in experiment 2. This study was conducted in accordance with the ethical guidelines of the Utsunomiya University Animal Experimentation Committee (Approval No. A22-0002) and was in complete compliance with the National Institutes of Health: Guide for the Care and Use of Laboratory Animals. All efforts were made to minimize the number of animals used and to limit experimentation to what was necessary to produce reliable scientific information.

#### 2.4.2. Procedure

We conducted the forced swim test to clarify the effects of the single compounds. In this experiment, mice were divided into six groups: five groups presented with several single compounds and a control group presented with triethyl citrate. We presented the mice with the following single compounds: Terpinen-4-ol, Hexanal, *trans*-2-Hexenal, Camphene, and *cis*-3-Hexenyl formate. These compounds are included in the SD but not in the CP. The forced swim test was conducted using the same methods and presentation conditions as experiment 2.

## 3. Results

### 3.1. Experiment 1: Antistress Effect of Fragrance under Mental Stress Load with Human Participants

**Figure 1:**
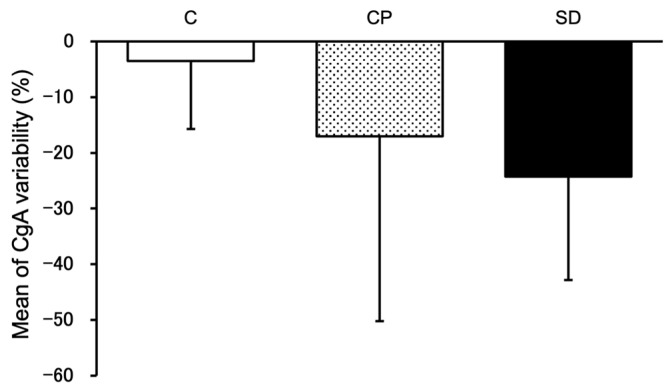
Mean of human chromogranin A (CgA) variability before and after performing the stress task. Yuzu synthetic fragrances had reduced tendencies of the human CgA variability (mean ± SEM, *n* = 10–12).

The rate of change in salivary CgA concentration was calculated from the CgA concentration in saliva collected immediately after the stress task, using the CgA concentration in saliva collected during the resting state as the standard (Figure 1). When the CgA change rate decreased, it was judged to have an antistress effect. Figure 1 shows that, although there was no significant difference, the variability rate in CgA decreased in the fragrance group compared to the control group. The SD group decreased by more than 20%.

**Figure 2:**
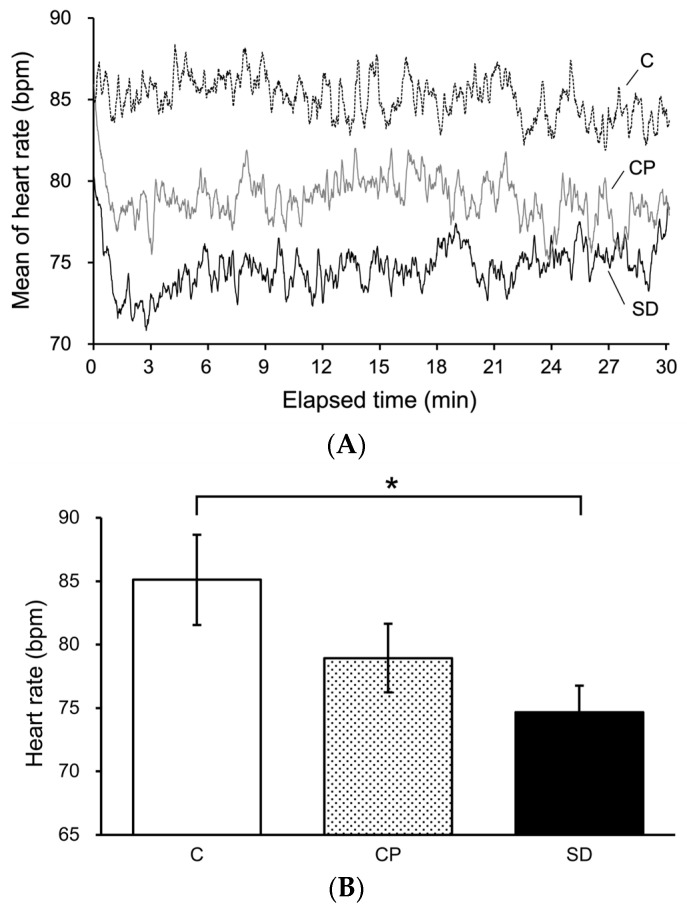
(**A**) Changes in mean of human heart rates (HRs) during the stress tasks. The horizontal axis presents the elapsed time from task onset. The vertical axis presents the changes in the mean HRs of all subjects in each group. Yuzu synthetic fragrances had reduced tendencies of the mean of human HRs. (**B**) Mean of human HRs during the stress task. Yuzu synthetic fragrances reduced the human HRs during the stress task (mean ± *SEM*, *n* = 10–13, * *p* < 0.05, Dunnet’s test vs. Control).

Figure 2A shows the mean HRs of each group. The *x*-axis is the elapsed time from the start of the calculation task and the *y*-axis is the HR. Figure 2B shows the mean HRs during the entire task response time for each group. Figure 2A shows that the mean HRs of the CP and SD groups were lower than that of the control group from immediately after the start of the task to the end of the task. Figure 2B shows that the HR of the SD group was significantly lower than that of the control group.

### 3.2. Experiment 2: Forced Swim Test Exposed to Synthetic Fragrances and Gene Expression Measurement in Mice

**Figure 3:**
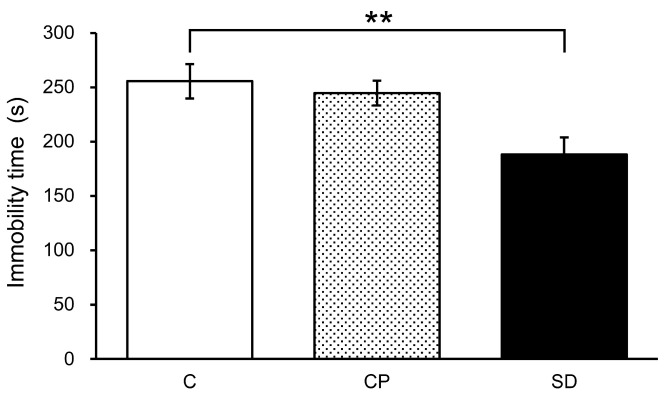
Antistress effect of synthetic fragrance mimicked steam-distilled oil (SD) on the forced swim test on mice. SD reduced immobility time (mean ± SEM, *n* = 7. ** *p* < 0.01, Dunnet’s test vs. Control).

We conducted the forced swim test twice on mice. In the first forced swim test, immobility time was found to have significantly decreased in the SD group compared to the control group (Figure 3).

**Figure 4:**
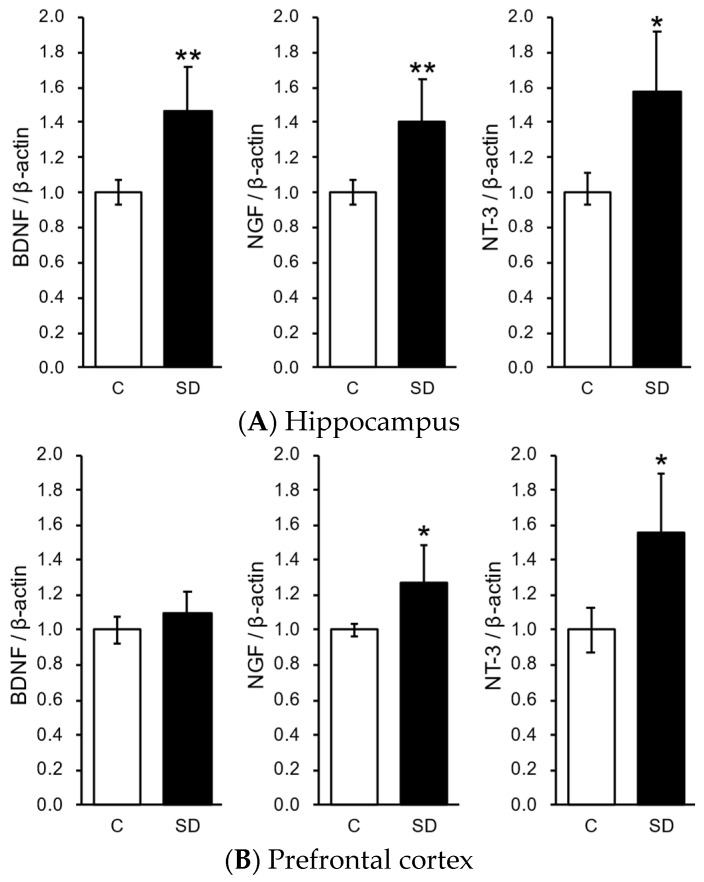
Antistress effect of Synthetic fragrance mimicked steam-distilled oil (SD) on the mRNA expression of neurotrophic and growth factors in the hippocampus and the prefrontal cortex of mice. (**A**) SD increased BDNF, NGF, and NT-3 mRNA expressions in the hippocampus (mean ± SEM, *n* = 8. ** *p* < 0.01, * *p* < 0.05, *t*-test vs. Control); (**B**) SD increased NGF and NT-3 mRNA expression in the prefrontal cortex (mean ± SEM, *n* = 8. * *p* < 0.05, *t*-test vs. Control).

After the second forced swim test, we investigated the effects of SD on the mRNA expression of neurotrophic and growth factors in the hippocampus and the prefrontal cortex. Hippocampal BDNF, NGF, and NT-3 mRNA expression were significantly stronger in the SD group than in the control group (Figure 4A). In addition, prefrontal cortex NGF and NT-3 mRNA expression were significantly stronger in the SD group than in the control group (Figure 4B).

### 3.3. Experiment 3: Forced Swim Test in Mice Exposed to a Single Compound

**Figure 5:**
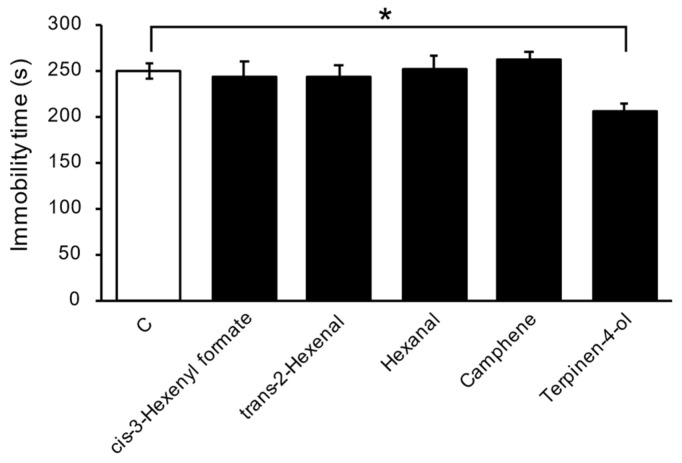
Antistress effect of terpinen-4-ol on the forced swim test on mice. Terpinen-4-ol reduced immobility time (mean ± SEM, *n* = 8. * *p* < 0.05, Dunnet’s test vs. Control).

In the forced swim test that exposed the mice to a single compound, immobility time was significantly lower in the terpinen-4-ol group compared to the control group (Figure 5).

## 4. Discussion

In experiment 1, the participants of the fragrance presentation group showed similar antistress effects on two antistress indices, CgA variability and HR. In both cases, the SD group showed greater antistress effects than the CP group. The naturally occurring monoterpene, terpinen-4-ol, exhibits a broad spectrum of pharmacological activities, including antimicrobial, antifungal, anti-inflammatory, and antioxidant properties [22,23,24]. In a study on pharmacological activity, Abbasi-Maleki et al. performed the forced swim test in mice intraperitoneally injected with the essential oil *Origanum majorana* to examine the antidepressant-like effects of the essential oil. Given that terpinen-4-ol is the primary constituent of the essential oil *Origanum majorana*, Abbasi-Maleki et al. suggested that terpinen-4-ol has antidepressant-like effects [25]. The presence of terpinen-4-ol in the SD fragrance and its absence in the CP fragrance suggest that terpinen-4-ol may exert antistress effects not only in mice but also in humans. Additionally, the concentration of linalool was slightly higher in the SD fragrance (2.20%) compared to the CP fragrance (1.40%), as shown in Table 1. Several studies on the antistress effects of linalool have been reported. Sugawara et al. analyzed the sedative effects of linalool using electroencephalography (EEG). As a result, the intensity of the beta wave of participants exposed to linalool decreased, suggesting that linalool has a sedative effect on humans [26]. Matsumoto et al. conducted an experiment on the antistress effects of natural yuzu flavoring using human CgA as an indicator. As a result, Matsumoto et al. reported a decrease in CgA concentration in subjects exposed to yuzu fragrance and identified linalool as one of the components that may have contributed to the decrease in CgA [9]. These reports suggest that the presence of terpinen-4-ol and the high concentration of linalool in the SD fragrance may contribute to its antistress effect on humans. Furthermore, it has been reported that the scent of limonene, the main component of citrus essential oils, has calming and antidepressant effects. Zhang also reported a significant recovery of decreased levels of monoamine neurotransmitters in the brain in response to stress in mice inhaling limonene [27]. Antidepressant supplementation is known to increase the content of monoamines in brain regions. In the fragrance preparations used in the experiments, limonene was present in similar proportions in both the CP and SD groups (53.4% and 53.3%, respectively). Limonene may have contributed to the observed antistress effect, which could explain the decreasing trend in CgA and HR in the CP and SD groups compared to the control group.

The forced swim test assesses potential antidepressant effects by observing stress-induced depressive behavior in mice [21]. In experiment 2, we evaluated the antistress effect of yuzu synthetic fragrances using the forced swim test on mice. According to the results, inhalation of SD significantly reduced immobility time, whereas inhalation of CP did not affect immobility time. The observed trend was consistent with the results from the human study in experiment 1. In addition, for the forced swim test in experiment 3, the antistress effects of five single compounds contained in SD but not in CP were verified by a forced swim test on mice. The results showed that inhalation administration of the terpinen-4-ol significantly decreased immobility time. A previous study employed forced swim tests to investigate the antistress effects of *Origanum majorana* essential oils. In this study, intraperitoneal injection of the essential oil was shown to be effective [25]. In contrast, our study utilized inhalation administration for SD fragrance and single terpinen-4-ol, which demonstrated their antistress effects. This result provides the first evidence of inhaled terpinen-4-ol’s antistress effects. Ueno et al. conducted forced swim tests in mice treated with inhalation of hexanal and reported that an antistress effect was observed [28]. *trans*-2-hexenal, called leaf aldehyde, is one of the main compounds of green leaf odor. Nakatomi et al. prepared an equivalent mixture of *trans*-2-hexenal and *cis*-3-hexenol to clarify the antidepressant-like effects of the green odor and conducted a forced swim test in mice exposed to the odor. *Cis*-3-hexenol is also a representative compound of the green odor. The results showed a significant decrease in immobility time and an antidepressant effect of the green odor containing *trans*-2-hexenal [29]. However, in the present experiment, there was no significant difference in immobility time for any single compounds, including hexanal and *trans*-2-hexenal. This suggests that these compounds may not be responsible for the antistress effects of the SD.

Neurotrophic and nerve growth factors, such as BDNF, NGF, and NT-3, regulate the proliferation and differentiation of neural progenitor cells and exert neuroprotective effects [30,31]. Previous studies reported that the gene expression of BDNF in the hippocampus decreased with chronic stress [32,33,34]. Furukawa-Hibi et al. and Deltheil et al. demonstrated that the antidepressant-like activity of antidepressants disappeared in BDNF knockout mice [35,36]. Deltheil et al. also reported that the intraventricular administration of BDNF exhibited antidepressant activity. NGF regulates neuronal growth and maturation. The gene expression of NGF decreased in the hippocampus of chronic stress model mice. Similarly, increased NT-3 gene expression induces differentiation of neural progenitor cells in the hippocampus, while deletion of NT-3 significantly inhibits differentiation of neural progenitor cells [37,38]. Based on these findings, BDNF, NGF, and NT-3 are regarded as major factors that control stress-induced depressive behavior through the proliferation of neural progenitor cells. In the present experiment, BDNF, NGF, and NT-3 mRNA expression levels in the hippocampus were higher after the inhalation administration of SD than in controls. These results suggest that SD promoted the expression of BDNF and NGF in the hippocampus and subsequently induced the proliferation of hippocampal neural progenitor cells. The decrease in immobility time and the increase in the expression of BDNF, NGF, and NT-3 mRNA demonstrate that SD suppresses stress-induced depressive behavior in mice.

In conclusion, we revealed significant antistress effects from fragrances that mimicked steam-distilled oil (SD) in studies of humans and mice. We then revealed significant antistress effects from single terpinen-4-ol in a mice study for the first time. This research gains significance considering the advantages of inhalation administration. Inhalation offers a minimally invasive approach, potentially reducing the burden on patients compared to oral or injectable routes. However, we could not confirm the antistress effects of single compounds such as terpinen-4-ol, linalool, and limonene in human studies. Further research is needed to investigate these single compounds. Additionally, it is assumed that the antistress effect was due to the effect of pure terpinen-4-ol or the synergistic effect of terpinen-4-ol and other compounds. Future research should address the synergistic and antagonistic effects of these multiple compounds, as well as the minimum effective dose of terpinen-4-ol.

## Figures and Tables

**Table 1:** Content rate of compounds of synthetic fragrance mimicked cold-pressed oil (CP) and steam-distilled oil (SD). N.D.: not detected.

Compounds	CAS RN^®^	Content Rate (Volume %)	
		CP	SD
Limonene	5989-27-5	53.400	53.300
γ-Terpinene	99-85-4	27.200	14.500
p-Cymene	99-87-6	2.500	11.500
ß-Myrcene	123-35-3	2.700	6.800
α-Pinene	80-56-8	6.200	6.300
Linalool	78-70-6	1.400	2.200
ß-Pinene	19870-74-7	2.400	1.100
Terpinolene	586-62-9	0.500	0.500
ß-Ocimene	13877-91-3	0.500	0.400
Terpinen-4-ol	562-74-3	N.D.	0.200
α-Terpineol	98-55-5	0.050	0.050
Hexanal	66-25-1	N.D.	0.040
*trans*-2-Hexenal	6728-26-3	N.D.	0.020
Camphene	79-92-5	N.D.	0.010
*cis*-3-Hexenyl formate	33467-73-1	N.D.	0.001
Octanal	124-13-0	0.007	N.D.
Thymol	89-83-8	0.060	N.D.
*trans*-ß-Farnesene	18794-84-8	0.005	N.D.
Ethyl Acetate	141-78-6	0.010	N.D.
Dipropylene Glycol	25265-71-8	3.068	3.079

**Table 2:** Primer sequences for real-time RT-PCR.

Genes		Sequences	Product Size (bp)	NCBI ID
Neurotrophic factor				
BDNF	sense	TCAGTTGGCCTTTGGATACC	85	NM_007540
	antisense	GCGGCAGATAAAAAGACTGC		
NGF	sense	CAGGCAGAACCGTACACAGA	91	NM_013609
	antisense	CTGTGTCAAGGGAATGCTGA		
NT-3	sense	TGCCGGAAGACTCTCTCAAT	87	NM_001164034
	antisense	CATCCACCATCTGTTTGGAA		
Housekeeping gene				
β-actin	sense	TGCTTCTAGGCGGACTGTTACTG	68	NM_007393
	antisense	CTGCGCAAGTTAGGTTTTGTCA		

## Data Availability

The original data presented in the study are openly available in FigShare at https://doi.org/10.6084/m9.figshare.26527933.v1 (accessed on 11 June 2024).

## Supplementary Materials

The following supporting information can be downloaded at: https://www.mdpi.com/article/10.3390/foods13193051/s1, Table S1: the retention indices (RI) data identified by GC/MS analysis; Figure S1: total ion chromatogram of the retention indices (RI) data identified by GC/MS analysis; Table S2: content rate of compounds of natural yuzu essential oils extracted by cold press; Table S3: content rate of compounds of natural yuzu essential oils extracted by steam distillation.

## Abbreviations

CgA	Chromogranin A
HR	Heart rate
GC/MS	Gas chromatography–mass spectrometry
CP	Synthetic fragrances mimicked cold-pressed oil
SD	Synthetic fragrances mimicked steam-distilled oil
BDNF	Primers specific for mouse brain-derived neurotrophic factor
NGF	Nerve growth factor
NT-3	Neurotrophin-3
