# Supplementary material for: Antistress Effects of Terpinen-4-ol and Compounds of Mimicked Yuzu Synthetic Fragrance in Humans and Mice
Source: Foods. 2024 Sep 25;13(19):3051. doi: 10.3390/foods13193051 (PMC11475151; doi:10.3390/foods13193051)
Supplement: Supplementary file 1 [file foods-13-03051-s001.zip › foods-3170404-supplementary.pdf]

September 12, 2024

Supplementary Materials

Antistress Effects of Terpinen-4-ol and Compounds of
Mimicked Yuzu Synthetic Fragrance in Humans and Mice

Takuma Kitamoto, Takafumi Mizushige, Xiaonan Xie, Taisei Uematsu,
Risako Ogura, Kakeru Sato, Yuki Yamazaki , Tsubasa Matsushita,
and Hiroshi Hasegawa

Table S1. The retention indices (RI) data identified by GC/MS analysis.

No	RT	RI	Area	Hit	CmdName	CAS No	Formula	MW	Library
1	3.867	854	1,199,369,673	91	Decane	124-18-5	C10H22	142.172	NIST17.L
2	4.913	929	837,579,078	95	Undecane	1120-21-4	C11H24	156.188	NIST17.L
3	6.279	1019	954,818,194	95	Dodecane	112-40-3	C12H26	170.203	NIST17.L
4	7.828	1097	1,124,257,510	97	Tridecane	629-50-5	C13H28	184.219	NIST17.L
5	9.438	1163	1,151,266,967	96	Tetradecane	629-59-4	C14H30	198.235	NIST17.L
6	11.032	1227	1,133,822,677	96	Pentadecane	629-62-9	C15H32	212.250	NIST17.L
7	12.569	1285	1,113,489,855	98	Hexadecane	544-76-3	C16H34	226.266	NIST17.L
8	14.044	1341	1,118,602,044	98	Heptadecane	629-78-7	C17H36	240.282	NIST17.L
9	15.451	1394	1,120,703,501	99	Octadecane	593-45-3	C18H38	254.297	NIST17.L
10	16.796	1447	1,108,583,375	98	Nonadecane	629-92-5	C19H40	268.313	NIST17.L
11	18.120	1498	1,718,900,943	99	Eicosane	112-95-8	C20H42	282.329	NIST17.L
12	19.326	1548	1,116,662,883	99	Heneicosane	629-94-7	C21H44	296.344	NIST17.L
13	20.509	1596	1,116,089,968	99	Docosane	629-97-0	C22H46	310.360	NIST17.L
14	21.648	1644	1,123,508,870	99	Tricosane	638-67-5	C23H48	324.376	NIST17.L
15	22.747	1691	1,137,230,946	99	Tetracosane	646-31-1	C24H50	338.391	NIST17.L
16	23.804	1738	1,126,961,637	95	Pentacosane	629-99-2	C25H52	352.407	NIST17.L
17	24.824	1784	1,143,461,125	99	Hexacosane	630-01-3	C26H54	366.423	NIST17.L
18	25.811	1829	1,148,454,551	99	Heptacosane	593-49-7	C27H56	380.438	NIST17.L
19	26.765	1874	1,141,454,553	92	Octacosane	630-02-4	C28H58	394.760	NIST17.L
20	27.704	1919	1,171,789,391	96	Nonacosane	630-03-5	C29H60	408.787	NIST17.L

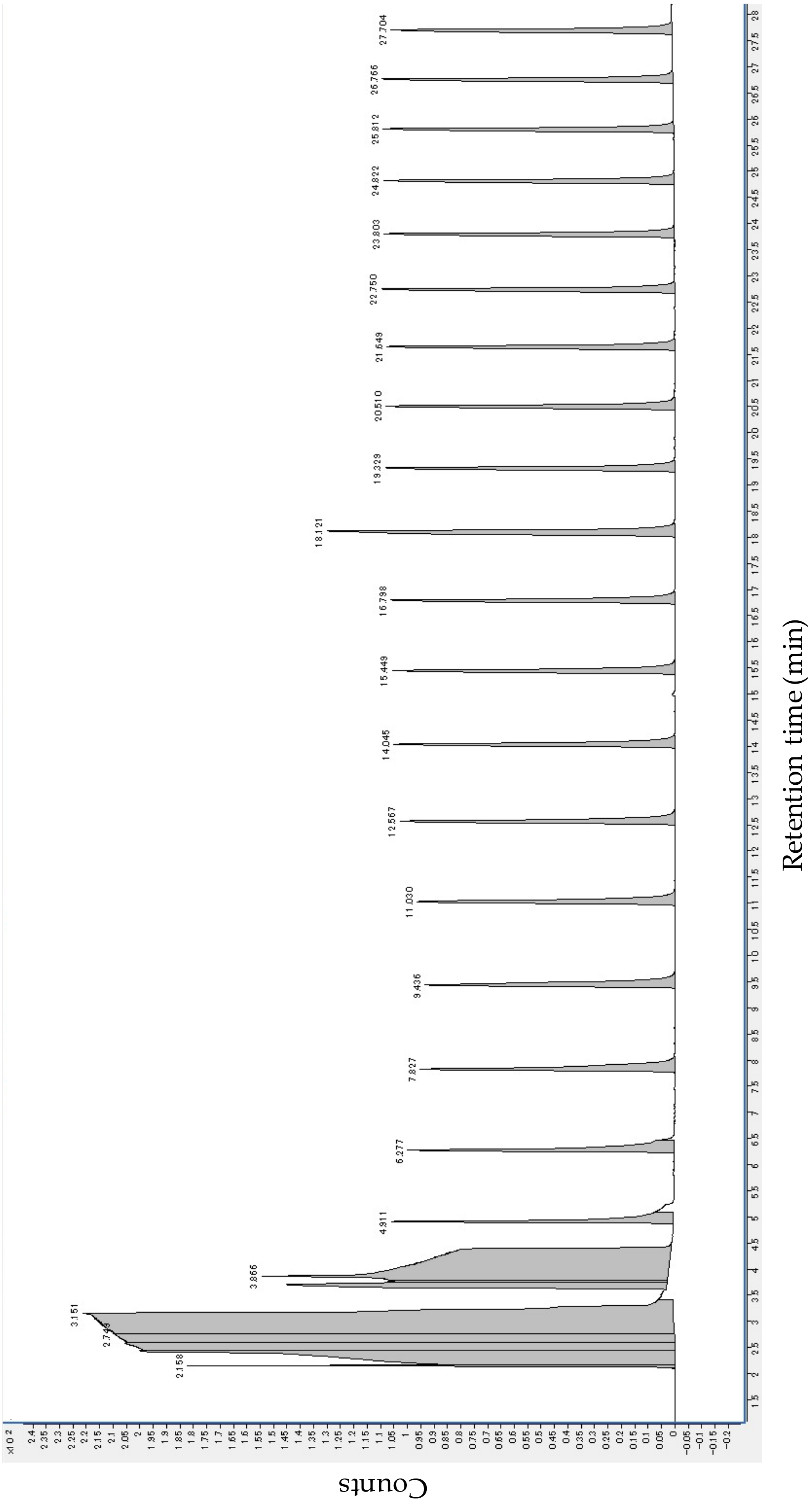
Retention time (min)
Counts
x10 2
2.158
3.151
3.866
4.311
6.277
7.827
9.436
11.030
12.567
14.045
15.449
16.798
18.121
19.329
20.510
21.649
22.750
23.803
24.822
25.812
26.766
27.704

Table S2. Content rate of compounds of natural yuzu essential oils extracted by cold-press.

Retention time (min)	Retention indices	Compounds	CAS RN®	Content rate (volume %)
3.5154	966	Ethyl Acetate	141-78-6	0.009
4.5507	1065	(1R)-2,6,6-Trimethylbicyclo[3.1.1]hept-2-ene	7785-70-8	6.216
4.9401	1102	Bicyclo[4.1.0]heptane, 7-methylene-	54211-14-2	0.003
5.0279	1108	Bicyclo[2.2.1]heptane, 7,7-dimethyl-2-methylene-	471-84-1	0.022
5.5221	1144	Bicyclo[3.1.1]heptane, 6,6-dimethyl-2-methylene-, (1S)-	18172-67-3	2.411
5.6862	1157	Bicyclo[3.1.0]hexane, 4-methylene-1-(1-methylethyl)-	3387-41-5	1.041
6.2206	1196	.beta.-Myrcene	123-35-3	2.641
6.2747	1069	.alpha.-Phellandrene	99-83-2	1.878
6.3410	1204	Bicyclo[2.2.2]oct-5-en-2-ol	55320-40-6	0.017
6.4827	1213	1,3-Cyclohexadiene, 1-methyl-4-(1-methylethyl)-	99-86-5	0.421
6.7982	1234	D-Limonene	5989-27-5	53.342
6.9148	1157	.beta.-Phellandrene	555-10-2	8.650
7.2614	1183	Bicyclo[3.1.0]hex-2-ene, 2-methyl-5-(1-methylethyl)-	2867-05-2	0.014
7.4616	1276	.gamma.-Terpinene	99-85-4	16.201
7.5271	1281	trans-.beta.-Ocimene	3779-61-1	0.424
7.8502	1301	o-Cymene	527-84-4	2.440
8.0380	1313	Cyclohexene, 1-methyl-4-(1-methylethylidene)-	586-62-9	0.488
8.1395	1319	Octanal	124-13-0	0.007
10.4651	1464	Benzene, 4-ethenyl-1,2-dimethyl-	27831-13-6	0.074
10.4657	1464	Benzene, 1-methyl-3-(1-methylethenyl)-	1124-20-5	0.075
10.8534	1489	Cyclohexanol, 1-methyl-4-(1-methylethenyl)-, cis-	7299-41-4	0.015
12.1289	1571	Linalool	78-70-6	1.358
13.0137	1630	3-Cyclohexen-1-ol, 4-methyl-1-(1-methylethyl)-, (R)-	20126-76-5	0.020
13.3509	1653	Propane, 1,3-dimethoxy-	17081-21-9	0.071
14.0199	1636	(E)-.beta.-Famesene	18794-84-8	0.005
14.3766	1723	.alpha.-Terpineol	98-55-5	0.054
14.7226	1748	cis-Muurola-4(15),5-diene	157477-72-0	0.010
15.0609	1772	Cyclohexane, 1-ethenyl-1-methyl-2-(1-methylethenyl)-4-(1-methylethylidene)-	3242-08-8	0.023
15.5699	1809	p-Mentha-1,5-dien-8-ol	1686-20-0	0.009
20.7121	2218	Thymol	89-83-8	0.061

Table S3. Content rate of compounds of natural yuzu essential oils extracted by steam-distillation.

Retention time (min)	Retention indices	Compounds	CAS RN®	Content rate (volume %)
4.5485	1065	(1R)-2,6,6-Trimethylbicyclo[3.1.1]hept-2-ene	7785-70-8	6.271
4.6732	1077	Butanoic acid, 2,2-dimethyl-	595-37-9	0.012
5.0302	1109	Camphene	79-92-5	0.004
5.1853	1120	Hexanal	66-25-1	0.041
5.5203	1144	Bicyclo[3.1.1]heptane, 6,6-dimethyl-2-methylene-, (1S)-	18172-67-3	1.055
5.6849	1157	Bicyclo[3.1.0]hexane, 4-methylene-1-(1-methylethyl)-	3387-41-5	0.246
6.0537	1069	.alpha.-Phellandrene	99-83-2	0.004
6.2206	1196	.beta.-Myrcene	123-35-3	6.825
6.2742	1069	.alpha.-Phellandrene	99-83-2	4.857
6.3399	1204	1,3-Methanopentalene, octahydro-	13913-22-9	0.051
6.4818	1213	1,3-Cyclohexadiene, 1-methyl-4-(1-methylethyl)-	99-86-5	1.560
6.7988	1234	D-Limonene	5989-27-5	53.321
6.9150	1157	.beta.-Phellandrene	555-10-2	3.856
7.0429	1249	2-Hexenal, (E)-	6728-26-3	0.018
7.2543	1183	Bicyclo[3.1.1]hept-2-ene, 3,6,6-trimethyl-	4889-83-2	0.021
7.4622	1276	.gamma.-Terpinene	99-85-4	4.217
7.5267	1281	trans-.beta.-Ocimene	3779-61-1	0.395
7.8498	1301	o-Cymene	527-84-4	11.465
8.0383	1313	Cyclohexene, 1-methyl-4-(1-methylethylidene)-	586-62-9	0.440
8.0400	1313	Benzene, (1-methoxypropyl)-	59588-12-4	1.299
8.1369	1319	3-Amino-1,2,4-triazole-5-carboxylic acid	3641-13-2	0.016
9.0081	1373	2-Propenal	107-02-8	0.013
9.5336	1406	3-Hexen-1-ol, formate, (Z)-	33467-73-1	0.001
9.7978	1423	Ethanone, 1-(2-methylphenyl)-	577-16-2	0.006
10.4659	1464	Benzene, 1-methyl-3-(1-methylethenyl)-	1124-20-5	0.236
10.5017	1467	Benzene, 1,4-diethyl-	105-05-5	0.046
12.1271	1571	Linalool	78-70-6	2.198
13.0118	1630	Terpinen-4-ol	562-74-3	0.145
13.3467	1653	Propane, 1,3-dimethoxy-	17081-21-9	0.062
14.3721	1723	.alpha.-Terpineol	98-55-5	0.045
20.7126	2218	Pentandioic acid, (p-t-butylphenyl) ester	212762-88-4	0.059
24.0359	2523	5-Hydroxy-4-octanone	496-77-5	0.035
